# Implementation of a constitutive model for anisotropic rocks based on modified Lade failure criterion

**DOI:** 10.1038/s41598-023-30257-z

**Published:** 2023-02-24

**Authors:** Xiaotian Liu, Yansheng Deng, Baoping Zou

**Affiliations:** 1 College of Civil Engineering, Tongji University, Shanghai, 200092 People’s Republic of China; 2 China Construction Eighth Engineering Division Corp., Ltd., Shanghai, 200112 People’s Republic of China; 3 School of Civil Engineering and Architecture, Zhejiang University of Science and Technology, Hangzhou, 310023 People’s Republic of China

**Keywords:** Petrology, Civil engineering

## Abstract

The strength anisotropy for inherent anisotropic rocks presents a challenge to simulate considering the influence of intermediate principal stress. Based on the modified Lade failure criterion, a new anisotropic modified Lade criterion combining an empirical equation is established and verified using published experimental datasets of anisotropic rocks. The incremental constitutive model for anisotropic rocks is derived by using finite difference theory. The dynamic link library (DLL) module of the model is obtained by using the VC++ program, and then the model is validated against various laboratory test results. It is concluded that the proposed criterion and derived model can describe the strength anisotropy of inherent anisotropic rocks well.

## Introduction

**Figure 1:**
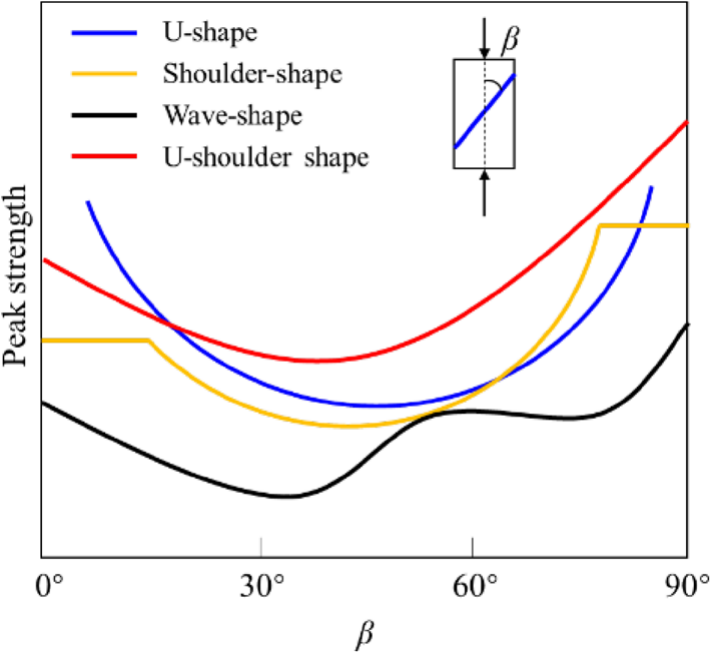
Strength characteristic curves of anisotropic rock with *β.*

Recently, some classical strength criteria for isotropic rocks in elastoplastic modeling have been continuously improved and applied by many researchers. Yao et al.^1^ proposed a generalized non-linear strength theory based on the experimental data and previous achievements. In order to determine the magnitude and direction of plastic strain increment, Lu et al.^2^ presented a three-dimensional fractional elastoplastic constitutive model for concrete material based on fractional derivative. Zhou et al.^3^ developed a plastic damage model with the non-orthogonal flow rule. Similarly, based on the non-orthogonal flow rule, Lu et al.^4^ proposed a cohesion-friction combined hardening plastic model, which was then implemented into ABAQUS using the implicit return mapping algorithm. Unlike isotropic rocks, anisotropic rocks such as slate, schist, gneisses, sandstone, and shale have a nonlinear strength response due to their inherent planes of anisotropy. Lots of research revealed that the loading direction *β* (angle between major principal direction and orientation of anisotropy plane) has a significant influence on the anisotropic strength. For most anisotropic rocks, the maximum strength has been noticed either at *β* = 0° or 90°, while the minimum strength is usually observed at *β* = 30°–45°^5–7^. Figure 1 presents the typical strength characteristic curves of anisotropic rock with different *β*. If only sliding failure is considered without non-sliding failure for anisotropic rock with a single weak plane, its strength curves often show U-shape^5,8^. In order to consider also non-sliding failure, Jaeger and Cook^9^ improved the above-mentioned failure criterion based on M-C, which leads to a shoulder-shaped strength characteristic curve with equal strength at both sides. However, this is not consistent with most of the experimental results^10^. When anisotropic rock contains more than one group of weak plane, the wave shaped strength curve is always followed. If the rock with higher anisotropy, its strength characteristic curve is U-shoulder shape. Based on these studies, the failure modes of inherent anisotropic rock fall into two basic types, the sliding failure, and the non-sliding failure. The sliding failure means that failure occurs along the inherent weak plane, while the failure occurred through the rock matrix is called non-sliding failure.

Due to the special strength characteristics of anisotropic rocks, the directional variation of the strength for anisotropic rocks has been widely investigated over the last few decades^8,11^. To describe the anisotropic strength, direct or indirect modifications are developed based on classical failure criteria such Mohr − Coulomb (M − C), Hoek − Brown (H − B) failure criterion, etc. The direct method is to introduce a new parameter into the original criterion and keep the material parameters constant in terms of their orientation^12–14^. The indirect modification is to replace material parameters (like *m* or *s* from H − B) of the failure criterion with an empirical formula that describes the relationship between strength and the orientation angle, which was extensively studied in previous research^15–20^. Thus, various empirical equations or approaches were proposed to describe the inherent anisotropy. Jaeger^8^ proposed the formula of cohesion of anisotropic rocks varied with the *β*. And later modified by Donath^5,21^. To describe the variation of the anisotropic strength with the orientation angle, *β*, McLamore and Gray^22^ improved the Donath’s equation. Hoek and Brown^16^ studied the relationship between the strength parameters, *m*_*b*_ and *s*, and the direction of loading. Pietruszczak and Mroz^18^ introduced a second-order microstructure tensor to reflect the inherent strength of anisotropy, and then Pietruszczak and Mroz^19^ proposed the critical plane approach to describe the direction-dependent strength for anisotropic rocks. Based on an extensive uniaxial test database, Shi et al.^20^ proposed a modified H–B failure criterion for anisotropic rocks, and the presented empirical equation of the uniaxial compressive strength (UCS) for any *β* was also verified to have excellent prediction capabilities. However, the above-mentioned researches do not consider the effect of the intermediate principal stress due to the limitation of the adopted failure criteria.

In this study, a modified Lade failure criterion for anisotropic rocks was proposed and verified based on an extensive uniaxial and triaxial test database at first. Then, the three-dimensional anisotropic modified Lade model is formulated in terms of stress and strain increments. The DLL module of the model is obtained by using the VC++ program based on the finite difference theory. At last, the modified Lade model for anisotropic rocks is validated against various laboratory test results.

## Problem description and basic equations

The Lade failure criterion was initially proposed by Lade for the failure of frictional materials, which describes the failure of materials using a special relationship between the first and the third invariants of the stress tensor as follows^23^:

1 $$(\frac{{I_{1}^{3} }}{{I_{3} }} - 27)(\frac{{I_{1} }}{{P_{a} }})^{k} = \eta$$

2 $$\begin{gathered} I_{1} = \sigma_{1} + \sigma_{2} + \sigma_{3} \hfill \\ I_{3} = \sigma_{1} \sigma_{2} \sigma_{3} \hfill \\ \end{gathered}$$

where *σ*_1_, *σ*_2_, and *σ*_3_ are the three principal stresses, *I*_1_ and *I*_3_ are the first and third stress tensor invariants, *k* and *η* are material constants, *P*_*a*_ is atmospheric pressure.

To consider the influence of the intermediate principal stress on wellbore stability in a simple methodology, the modified Lade failure criterion was proposed by Ewy^24^, which is a simplified version of the Lade failure criterion. Similar to the M–C failure criterion, the modified Lade criterion is only governed by two most commonly used parameters: *c* and *φ*, which can be written as follows:

3 $$\frac{{(I_{1}^{^{\prime\prime}} )^{3} }}{{I_{3}^{^{\prime\prime}} }} = 27 + \eta$$

4 $$\begin{gathered} I_{1}^{^{\prime\prime}} = (\sigma_{1} + S_{a} - P_{p} ) + (\sigma_{2} + S_{a} - P_{p} ) + (\sigma_{3} + S_{a} - P_{p} ) \hfill \\ I_{3}^{^{\prime\prime}} = (\sigma_{1} + S_{a} - P_{p} )(\sigma_{2} + S_{a} - P_{p} )(\sigma_{3} + S_{a} - P_{p} ) \hfill \\ \end{gathered}$$

where $I_{1}^{^{\prime\prime}}$ and $I_{3}^{^{\prime\prime}}$ are the appropriate first and third stress tensor invariants. *S*_*a*_ and *η* are material constants. *P*_*p*_ is pore pressure. *S*_*a*_ represents the cohesion of rock, *c*, while *η* is determined by internal friction angle, *φ*. Calculations show that both *S*_*a*_ and *η* can be obtained directly from the M–C criterion *c* and *φ* by

5 $$S_{a} = \frac{c}{\tan \varphi }$$

6 $$\eta { = }4{\text{tan}}^{{2}} \varphi \frac{9 - 7\sin \varphi }{{1 - \sin \varphi }}$$

where *c* and *φ* can be obtained by the conventional triaxial compression tests.

For anisotropic rocks such as slate, gneisses, schist, and shale, the strength highly depends on the loading direction^7,25–27^. To predict the strength of anisotropic rocks, an indirect modification method with empirical function is widely used to modify the material strength parameters based on the orientation angle, *β*^8,15,16^. That means the strength parameters *c* and *φ* are orientation-dependent which needs assignment for each *β*. Thus, the anisotropic modified Lade failure criterion can be given by

7 $$\frac{{(I_{1\beta }^{^{\prime\prime}} )^{3} }}{{I_{3\beta }^{^{\prime\prime}} }} = 27 + \eta_{\beta }$$

8 $$\begin{gathered} I_{1\beta }^{^{\prime\prime}} = (\sigma_{1} + S_{a\beta } - P_{p} ) + (\sigma_{2} + S_{a\beta } - P_{p} ) + (\sigma_{3} + S_{a\beta } - P_{p} ) \hfill \\ I_{3\beta }^{^{\prime\prime}} = (\sigma_{1} + S_{a\beta } - P_{p} )(\sigma_{2} + S_{a\beta } - P_{p} )(\sigma_{3} + S_{a\beta } - P_{p} ) \hfill \\ \end{gathered}$$

9 $$\eta_{\beta } { = }4{\text{tan}}^{{2}} \varphi (\beta )\frac{9 - 7\sin \varphi (\beta )}{{1 - \sin \varphi (\beta )}}$$

10 $$S_{a\beta } = \frac{c(\beta )}{{\tan \varphi (\beta )}}$$

Based on an extensive triaxial test database, the maximum UCS of anisotropic rocks is at *β* = 90°, while the other is at *β* = 0°. To figure out this discrepancy, Shi et al.^20^ proposed a new empirical equation of the UCS to describe the relationship between material parameters (*c* and *φ*) and *β* as follows:

11 $$\left\{ \begin{gathered} \gamma (\beta ) = \gamma_{0} - (\gamma_{0} - \gamma_{\min } )\left[ {sin(\frac{90^\circ \beta }{\theta })} \right]^{m} ,\quad 0^\circ \le \beta \le \theta \hfill \\ \gamma (\beta ) = \gamma_{90} - (\gamma_{90} - \gamma_{\min } )\left[ {cos(\frac{90^\circ (\beta - \theta )}{{90^\circ - \theta }})} \right]^{n} ,\quad \theta \le \beta \le 90^\circ \hfill \\ \end{gathered} \right.$$

where *γ*_min_ is the minimum value of strength parameter, *γ*_0_ and* γ*_90_ are strength parameters at *β* = 0° and 90°,* m* and *n* are constants, and *θ* is the angle corresponding to the minimum strength parameter.

Among the aforementioned parameters, *γ*_0_,* γ*_90_, *γ*_min_, *m*, *n*, and *θ* are the anisotropic parameters of the proposed model, which can capture the strength anisotropy. *γ*_0_, *γ*_90_, *γ*_min_ and *θ* can be determined directly by test results. To calibrate the remaining two parameters (*m* and *n*), the trial-and-error method can be employed to obtain the optimal solutions based on the least sum of square error (LSSE). Take the UCS of layered rock as an example provided by Zhang et al.^28^. To cohesive, the *γ*_0_, *γ*_90_, *γ*_min_ and *θ* are 24.84 MPa, 28.97 MPa, 19.95 MPa and 35°, respectively, based on the test data. Substituting them into Eq. (11), *m* and *n* can be determined as 1.8 and 2.1 (LSSE is 8.0e−4) based on trial-and-error method and LSSE. Generally, the trial-and-error method for *m* and *n* can start with a smaller integer, like 1, and then increase gradually with the interval of 0.1.

The prediction capacities of the developed anisotropic modified Lade failure criterion were analyzed and verified by three different error measures, percent error (PE) for each data point, coefficient of accordance (COA), and average absolute relative error percentage (AAREP)^29,30^, which are given by

12 $$PE = \left( {\frac{{\sigma_{{{\text{1cal}}}} - \sigma_{1\exp } }}{{\sigma_{1\exp } }}} \right) \times 100\%$$

13 $$COA = \frac{{\sum {(\sigma_{1\exp } - \sigma_{{1{\text{cal}}}} )^{2} } }}{{\sum {(\sigma_{1\exp } - \sigma_{{1{\text{av}}}} )^{2} } }}$$

14 $$AAREP = \frac{{\sum\limits_{i = 1}^{N} {\left| {\frac{{\sigma_{{{\text{1cal}}}} - \sigma_{1\exp } }}{{\sigma_{1\exp } }}} \right|} }}{N} \times 100\%$$

where *σ*_1cal_ and *σ*_1exp_ are the experimental and predicted value of the major principal stress at failure for given confining pressure, *σ*_1av_ is the average of the experimental *σ*_1_ for the triaxial data set under consideration, *N* is the total number of triaxial data points.

**Figure 2:**
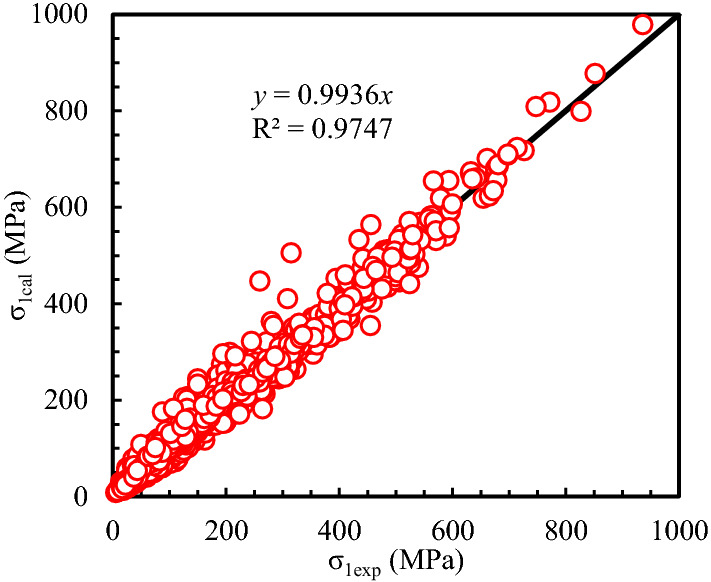
Predicted and Experimental *σ*_1_.

**Figure 3:**
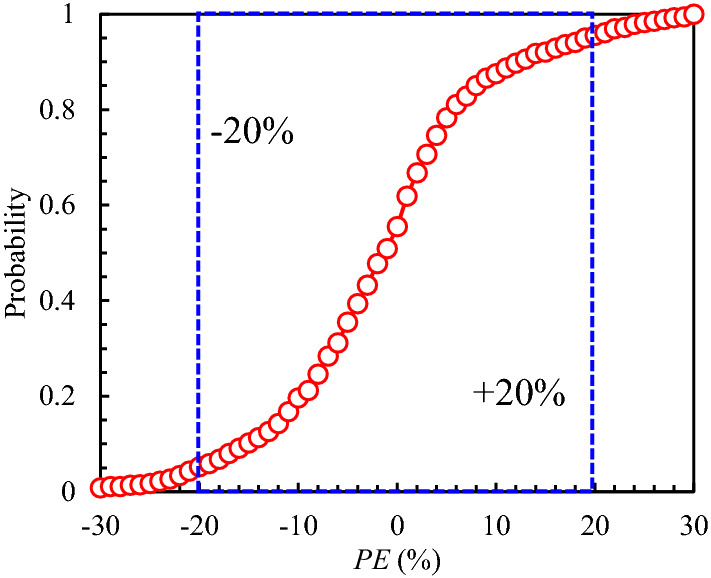
Probability distribution for percent error.

**Figure 4:**
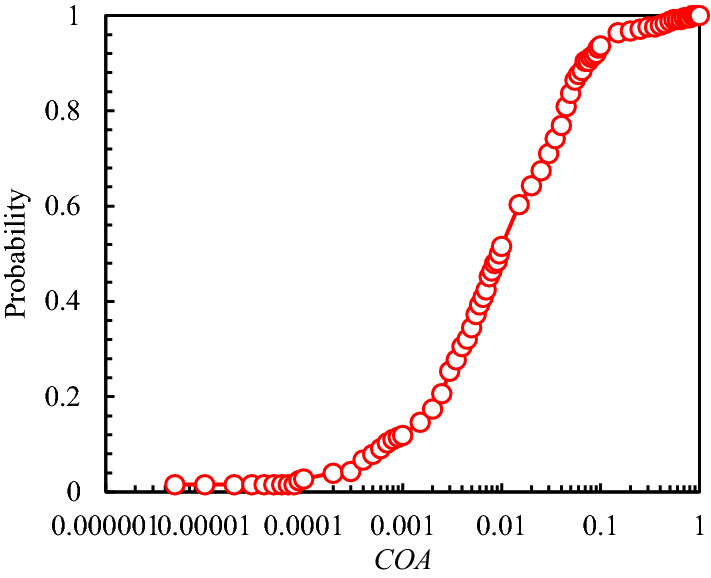
Probability distribution for COA.

An extensive database^30^ obtained from the uniaxial and triaxial tests on anisotropic rocks such as slate, shale, schist, sandstone, gneiss, phyllite, etc., were used to verify the prediction capacity. The predicted and experimental *σ*_1_ values were presented in Fig. 2, from which all points are closely scattered about a line with a gradient of 1:1. The regression coefficient, *R*^2^ is about 0.975, which means a good regression result. Figure 3 plots the probability distribution for PE based on Eq. (12) From Fig. 3, the probability of the PE to be within −20% to + 20% is about 0.91. The probability distribution for COA is plotted in Fig. 4. The probability of COA being less than or equal to 0.1 is 0.9354 which is considerably high. In addition, the AAREP is obtained to be 11.4%, which is also relatively low. Therefore, the proposed anisotropic modified Lade failure criterion fits well not only the uniaxial tests but also the triaxial in the provided database.

## Methods

The constitutive model formulation in FLAC^3D^ can be expressed as incremental form with the principal stresses *σ*_1_, *σ*_2_, *σ*_3_, and the principal strains *ɛ*_1_, *ɛ*_2_, *ɛ*_3_. Assuming that the compressive stress is positive, and $\overline{\sigma }_{i} = \sigma_{i} + S_{a} \,,\quad i = 1,2,3$, Based on the stress tensor invariants and the partial stress tensor invariants equation (see Supplementary Appendix), the anisotropic modified Lade failure criterion can be rewritten as

15 $$I_{1}^{3} - \alpha I_{3}^{{}} = 0$$

where *α* = 27 + *η*.

Substituting the relationship between stress tensor and invariants (see Supplementary Appendix) into Eq. (15) gives

16 $$(\frac{{I_{1} }}{{\sqrt {J_{2} } }})^{3} - \frac{{9\alpha I_{1} }}{{(\alpha - 27)\sqrt {J_{2} } }} - \frac{{18\alpha \sin (3\theta_{{\overline{\sigma } }} )}}{\sqrt 3 (\alpha - 27)} = 0,\quad \alpha > 27$$

Substituting $I_{1} /\sqrt {J_{2} } = r\sin \beta$ into Eq. (16) leads to

17 $$\sin^{3} \beta - \frac{9\alpha }{{r^{2} (\alpha - 27)}}\sin \beta - \frac{{18\alpha \sin (3\theta_{{\overline{\sigma } }} )}}{{\sqrt 3 r^{3} (\alpha - 27)}} = 0,\quad \alpha > 27$$

The relationship between different angles of sine function can be obtained as follows:

18 $$\sin^{3} \beta - \frac{3}{4}\sin \beta + \frac{1}{4}\sin (3\beta ) = 0\quad$$

Comparing Eq. (16) and Eq. (17), it can be seen that

19 $$\left\{ \begin{gathered} r = 2\sqrt {\frac{3\alpha }{{\alpha - 27}}} \hfill \\ \sin (3\beta ) = - \sqrt {\frac{\alpha - 27}{\alpha }} \sin (3\theta_{{\overline{\sigma } }} ) \hfill \\ \end{gathered} \right.$$

Therefore, Eq. (18) is a cubic equation with one variable, and its three roots are

20 $$\left\{ \begin{gathered} (\frac{{I_{1} }}{{\sqrt {J_{2} } }})_{1} = r\sin \beta = - \frac{2\sqrt 3 }{T}\sin (\frac{1}{3}arcsin(Tsin(3\theta_{{\overline{\sigma } }} ))) \hfill \\ (\frac{{I_{1} }}{{\sqrt {J_{2} } }})_{2} = r\sin (\beta - \frac{2\pi }{3}) = - \frac{2\sqrt 3 }{T}\sin (\frac{\pi }{3} - \frac{1}{3}arcsin(Tsin(3\theta_{{\overline{\sigma } }} ))) \hfill \\ (\frac{{I_{1} }}{{\sqrt {J_{2} } }})_{3} = r\sin (\beta + \frac{2\pi }{3}) = \frac{2\sqrt 3 }{T}\sin (\frac{\pi }{3} + \frac{1}{3}arcsin(Tsin(3\theta_{{\overline{\sigma } }} ))) \hfill \\ \end{gathered} \right.$$

where $T = \sqrt {(\alpha - 27)/\alpha }$. From Eq. (20), the first root is less than or equal to 0 in the range of 0 to π/6, the second root is always less than 0 between -π/6 and π/6. Only the third root is more than or equal to 0 in the same range. Therefore, the third root is used as the solution of Eq. (20), which can be written as

21 $$\left\{ \begin{gathered} \sqrt {J_{2} } = \frac{{TI_{1} }}{2\sqrt 3 }g(\theta_{{\overline{\sigma } }} ) \hfill \\ g(\theta_{{\overline{\sigma } }} ){ = }\frac{1}{{\sin (\frac{\pi }{3} + \frac{1}{3}arcsin(Tsin(3\theta_{{\overline{\sigma } }} )))}},\quad - \frac{\pi }{6} \le \theta_{{\overline{\sigma } }} \le \frac{\pi }{6} \hfill \\ \end{gathered} \right.$$

Referring to the form of generalized Mises strength criterion, Eq. (21) can be rewritten into a yield function

22 $$f^{s} = \sqrt {J_{2} } - 3m\overline{\sigma }$$

where *m* and *σ* can be expressed as

23 $$\left\{ \begin{gathered} m = \frac{{Tg(\theta_{{\overline{\sigma } }} )}}{2\sqrt 3 } \hfill \\ \sigma = \frac{1}{3}I_{1} \hfill \\ \end{gathered} \right.$$

The total strain increment *Δɛ*_*i*_ consists of elastic strain increment *Δɛ*_*i*_^*e*^ and plastic strain increment *Δɛ*_*i*_^*p*^ (with *i* = 1, 2, 3). Based on Hooke’s law, the principal stress increments can be expressed as

24 $$\left\{ \begin{gathered} \Delta \sigma_{1} = S_{1} (\Delta \varepsilon_{1}^{e} ,\Delta \varepsilon_{2}^{e} ,\Delta \varepsilon_{3}^{e} ) = \alpha_{1} \Delta \varepsilon_{1}^{e} + \alpha_{2} (\Delta \varepsilon_{2}^{e} + \Delta \varepsilon_{3}^{e} ) \hfill \\ \Delta \sigma_{2} = S_{2} (\Delta \varepsilon_{1}^{e} ,\Delta \varepsilon_{2}^{e} ,\Delta \varepsilon_{3}^{e} ) = \alpha_{1} \Delta \varepsilon_{2}^{e} + \alpha_{2} (\Delta \varepsilon_{1}^{e} + \Delta \varepsilon_{3}^{e} ) \hfill \\ \Delta \sigma_{3} = S_{3} (\Delta \varepsilon_{1}^{e} ,\Delta \varepsilon_{2}^{e} ,\Delta \varepsilon_{3}^{e} ) = \alpha_{1} \Delta \varepsilon_{3}^{e} + \alpha_{2} (\Delta \varepsilon_{1}^{e} + \Delta \varepsilon_{2}^{e} ) \hfill \\ \end{gathered} \right.$$

where *α*_1_ and *α*_2_ are material parameters, which can be given by

25 $$\left\{ \begin{gathered} \alpha_{1} = K + \frac{4}{3}G \hfill \\ \alpha_{2} = K - \frac{2}{3}G \hfill \\ \end{gathered} \right.$$

The plastic strain increment *Δɛ*_*i*_^*p*^ can be expressed by the flow rule

26 $$\Delta \varepsilon_{i}^{p} = \lambda \frac{{\partial g^{s} }}{{\partial \sigma_{i} }}$$

where *λ* is the plastic coefficient, *g* is the plastic potential function, which can be expressed by

27 $$g^{s} = \sqrt {J_{2} } - 3m\overline{\sigma }$$

Then

28 $$\left\{ \begin{gathered} \frac{{\partial g^{s} }}{{\partial \sigma_{1} }} = \frac{{2\sigma_{1} - \sigma_{2} - \sigma_{3} }}{{6\sqrt {J_{2} } }} - m \hfill \\ \frac{{\partial g^{s} }}{{\partial \sigma_{2} }} = \frac{{2\sigma_{2} - \sigma_{1} - \sigma_{3} }}{{6\sqrt {J_{2} } }} - m \hfill \\ \frac{{\partial g^{s} }}{{\partial \sigma_{3} }} = \frac{{2\sigma_{3} - \sigma_{1} - \sigma_{2} }}{{6\sqrt {J_{2} } }} - m \hfill \\ \end{gathered} \right.$$

Combining with Eqs. (26) and (27) gives

29 $$\Delta \varepsilon_{i}^{e} = \Delta \varepsilon_{i} - \lambda \frac{{\partial g^{s} }}{{\partial \sigma_{i} }}$$

Substituting Eq. (29) into Eq. (24) leads to

30 $$\Delta \sigma_{i} = S_{i} (\Delta \varepsilon_{n} ) - \lambda S_{i} (\frac{{\partial g^{s} }}{{\partial \sigma_{n} }}),\quad n = 1,2,3$$

The new stress state *σ*_*i*_^*N*^ corresponding to the total strain increment can be expressed by

31 $$\sigma_{i}^{N} = \sigma_{i} + \Delta \sigma_{i}$$

The induced elastic stresses (elastic guess) *σ*_*i*_^*I*^ can be given by

32 $$\sigma_{i}^{I} = \sigma_{i} + S_{i} (\Delta \varepsilon_{n} ),\quad n = 1,2,3$$

Substituting Eqs. (28), (30) and (32) into Eq. (31) gives

33 $$\left\{ \begin{gathered} \sigma_{1}^{N} = \sigma_{1}^{l} - \left[ {\alpha_{1} \lambda^{s} \left( {\frac{{2\sigma_{1} - \sigma_{2} - \sigma_{3} }}{{6\sqrt {J_{2} } }} - m} \right) + \alpha_{2} \lambda^{s} \left( {\frac{{\sigma_{2} + \sigma_{3} - 2\sigma_{1} }}{{6\sqrt {J_{2} } }} - 2m} \right)} \right] \hfill \\ \sigma_{2}^{N} = \sigma_{2}^{l} - \left[ {\alpha_{1} \lambda^{s} \left( {\frac{{2\sigma_{2} - \sigma_{1} - \sigma_{3} }}{{6\sqrt {J_{2} } }} - m} \right) + \alpha_{2} \lambda^{s} \left( {\frac{{\sigma_{1} + \sigma_{3} - 2\sigma_{2} }}{{6\sqrt {J_{2} } }} - 2m} \right)} \right] \hfill \\ \sigma_{3}^{N} = \sigma_{3}^{l} - \left[ {\alpha_{1} \lambda^{s} \left( {\frac{{2\sigma_{3} - \sigma_{1} - \sigma_{2} }}{{6\sqrt {J_{2} } }} - m} \right) + \alpha_{2} \lambda^{s} \left( {\frac{{\sigma_{1} + \sigma_{2} - 2\sigma_{3} }}{{6\sqrt {J_{2} } }} - 2m} \right)} \right] \hfill \\ \end{gathered} \right.$$

From Eq. (33),

34 $$\left\{ \begin{gathered} \sigma^{N} = \sigma^{l} + 3mK\lambda^{s} \hfill \\ \sqrt {J_{2} }^{N} = \sqrt {J_{2} }^{l} - G\lambda^{s} \hfill \\ \end{gathered} \right.$$

Substituting Eq. (34) into Eq. (22) gives

35 $$\lambda^{s} = \frac{{\sqrt {J_{2} }^{l} - 3m\sigma^{l} }}{{G + 9Km^{2} }}$$

The yield function of tensile stress *f*^*t*^ can be expressed by

36 $$f^{t} = \sigma - \sigma^{t}$$

Based on the associated flow rule, the potential function of tensile stress is

37 $$g^{t} = \sigma$$

According to Eqs. (37) and (38) can be obtained

38 $$\left\{ \begin{gathered} \frac{{\partial g^{t} }}{{\partial \sigma_{1} }} = \frac{1}{3} \hfill \\ \frac{{\partial g^{t} }}{{\partial \sigma_{2} }} = \frac{1}{3} \hfill \\ \frac{{\partial g^{t} }}{{\partial \sigma_{3} }} = \frac{1}{3} \hfill \\ \end{gathered} \right.$$

Substituting Eq. (38) into Eq. (36) gives

39 $$\left\{ \begin{gathered} \sigma_{1}^{N} = \sigma_{1}^{l} - \frac{1}{3}(\alpha_{1} \lambda^{t} + 2\alpha_{2} \lambda^{t} ) \hfill \\ \sigma_{2}^{N} = \sigma_{1}^{l} - \frac{1}{3}(\alpha_{1} \lambda^{t} + 2\alpha_{2} \lambda^{t} ) \hfill \\ \sigma_{3}^{N} = \sigma_{1}^{l} - \frac{1}{3}(\alpha_{1} \lambda^{t} + 2\alpha_{2} \lambda^{t} ) \hfill \\ \end{gathered} \right.$$

where

40 $$\lambda^{t} = \frac{{f^{t} }}{K} = \frac{{\sigma^{l} - \sigma^{t} }}{K}$$

$H(\sigma ,\sqrt {J_{2} } ) = 0$ is designed to determine the flow rule, which can be expressed by

41 $$H = \sqrt {J_{2} } - 3m\sigma^{t} - (\sqrt {1 + 9m^{2} } )(\sigma - \sigma^{t} )$$

**Figure 5:**
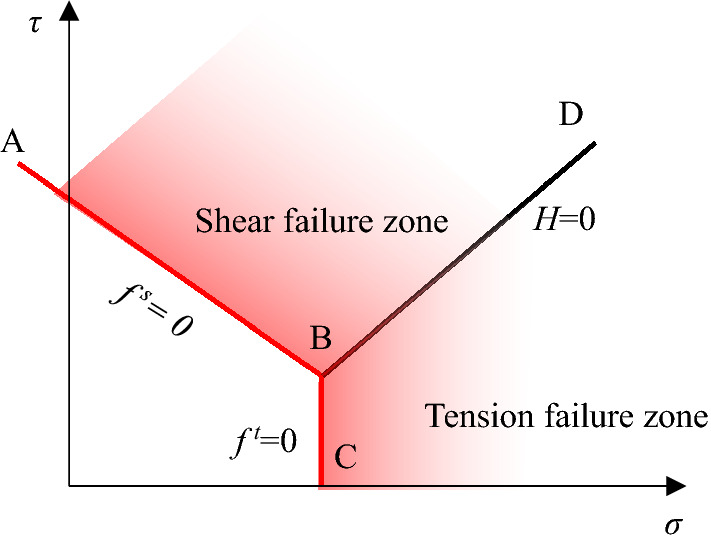
Modified Lade model domains used in the definition of the flow rule.

The composite modified Lade model domains used in the definition of the flow rule are shown in Fig. 5. The failure envelope *f*^s^ = 0 is defined by the modified Lade model failure criterion from point A to B on Fig. 5, and by the tension failure criterion *f*^*t*^ = 0 from B to C. The diagonal line (from B to D) between the representation of *f*^s^ = 0 and *f*^*t*^ = 0 in the (τ, σ) plane, divides the domain of the elastic guess violating the composite yield function into two, shear failure zone and tension failure zone, respectively. If *f*^*s*^ > 0 and *H* > 0, shear failure is declared, while tensile failure takes place if *f*^*t*^ > 0 and *H* < 0.

**Figure 6:**
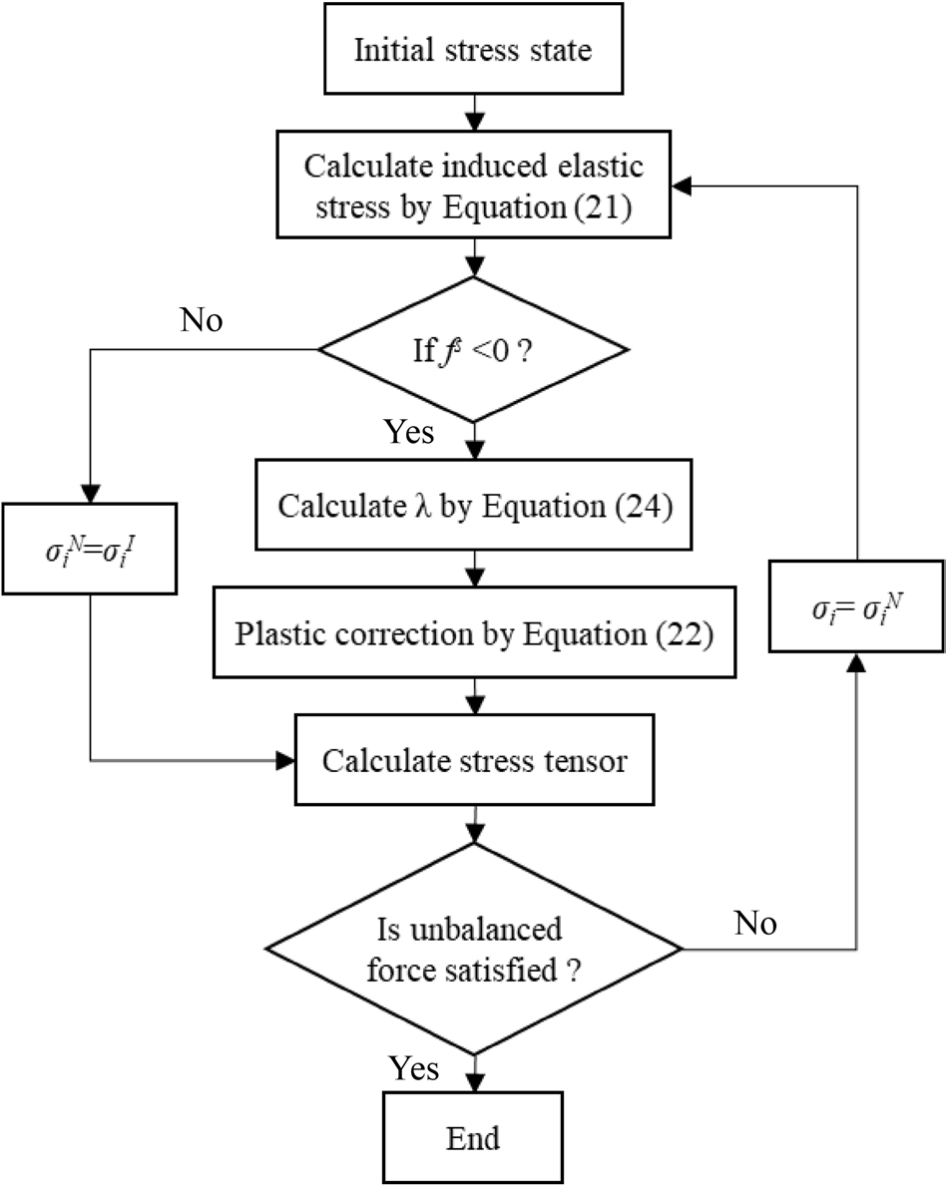
Flowchart of proposed model.

Figure 6 presents the flowchart of the proposed anisotropic modified Lade failure criterion. To apply the failure criterion to numerical simulation, a DLL module was programmed by compiling a program with C++ language, which can be read directly by FLAC^3D^. There are two simple appraochs for implementing constitutive models within FLAC^3D^ with plastic correction method^31^. In this work, an explicit form of the integration scheme of constitutive models provided by FLAC^3D^ can be utilized. A general yield criterion based on the anisotropic modified Lade failure criterion is employed, and the plastic coefficients for shear and tension can be calculated by setting the consistency condition, *g*^*s*^ = 0 and *g*^*t*^ = 0. The implementation process of the proposed model into FLAC^3D^ is as follows:

1. Initialize the model parameters, which is only executed once;
2. Obtain the total strain increment (*Δε*_*n*_), which includes the elastic and plastic components (*Δε*^*e*^_*n*_ and *Δε*^*p*^_*n*_);
3. Calculate elastic guess *σ*^*I*^ at time *t* + *Δt* using incremental elastic stress–strain law (Eq. (32)). In Eq. (32), *S*_*i*_(*Δε*_*n*_) is the component *i* of the stress increment induced by the total-strain increment *Δε*_*n*_, in case no increment of plastic deformation takes place. If the *σ*^*I*^ violates the yield function (Eq. (27) or Eq. (36)), Eq. (33) or Eq. (39) are used to place the new stress on the yield curve. Othewise, *σ*^*I*^ gives the new stress state at time *t* + *Δt*;
4. If the *σ*^*I*^ is located above the yield surface in the generalized stress space, *λ*^*s*^ or *λ*^*t*^ are calculated in Eq. (35) or Eq. (40), and then the new stress can be obtain based on Eq. (34) or Eq. (39);
5. Deliver new stress to FLAC^3D^.

## Numerical results and discussion

After generating the DLL module, lots of simulations (uniaxial and triaxial compressive tests of anisotropic rocks) with the user-defined anisotropic modified Lade model were conducted to verify the correctness of the proposed model. A cylindrical rock specimen with a 100 mm height and 50 mm diameter is generated, the normal load is applied to the top, and the surface load of circumferential normal is applied to present the confining pressure. The uniaxial and triaxial test database of four different types of anisotropic rocks is used for model validation (Table 1).

**Table 1:** Uniaxial and triaxial test database for model validation.

References	Rock type	*σ*_3_ (MPa)	*β* (°)
Niandou et al.^33^	Tournemire shale	0, 5, 20, 40	0, 30, 45, 60, 90
Tien and Tsao^34^	Artificial interlayered rock	0, 3, 8, 15	0, 15, 30, 45, 60, 75, 90
Tien and Tsao^34^	Transversely isotropic rock	0, 6, 14, 35	0, 15, 30, 45, 60, 75, 90
Zhang et al.^28^	Layered rock	0, 10, 20, 30	0, 15, 35, 70, 90

**Figure 7:**
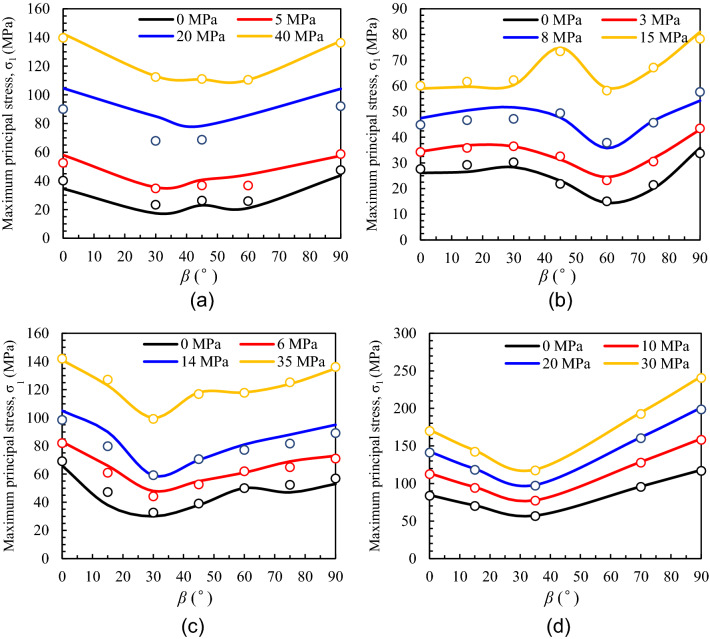
Comparison between numerical and experimental results: (**a**) Tournemire shale, (**b**) artificial interlayered rock, (**c**) transversely isotropic rock, (**d**) layered rock (the scattered points and solid lines represent the experimental and numerical results, respectively).

A comparison between numerical and experimental results is shown in Fig. 7. The experimental results are presented as scattered points while the numerical data are plotted by solid lines. It can be seen from Fig. 7 that the shape of strength variation of different anisotropic rocks with *β* presents a typical U-type. Except for artificial interlayered rock, the minimum compressive strength is seen at *β* = 30°–45°, while the maximum strength is shown at *β* = 0° or 90°. In summary, the compressive strength predicted is consistent with the lab data of the tested anisotropic rocks under different confining stresses, indicating the rationality and correctness of the proposed model.

**Figure 8:**
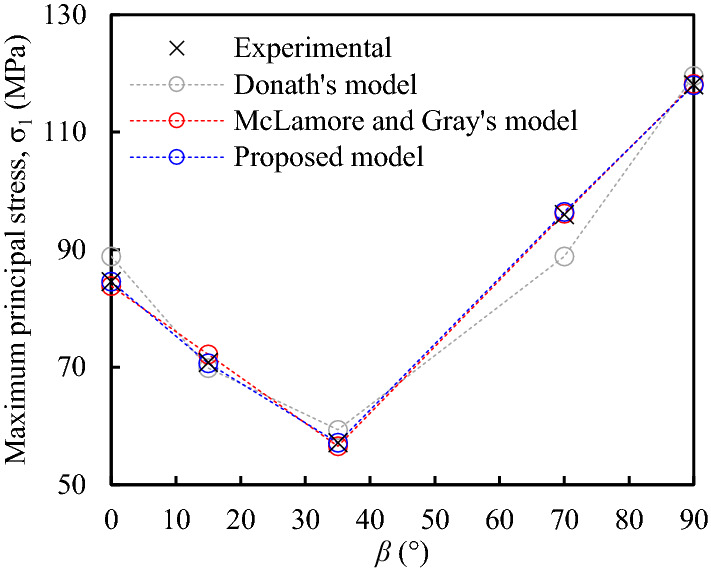
Comparison results between different model and experimental data.

In order to reflect the advantages of proposed model in describing the rock anisotropy, a group of comparison including Donath’s model, McLamore and Gray’s model, and proposed model, is carried out based on the experimental data of UCS^28^, which is shown in Fig. 8. As can be seen, the error of Donath’s model is relatively large. Both the McLamore and Gray’s model and proposed model can well capture the anisotropy for anisotropic rock, especially at *β* = 0°, 90°, and the angle corresponding to the minimum strength. In comparison, the proposed model is more accurate in reflecting the rock anisotropy.

**Figure 9:**
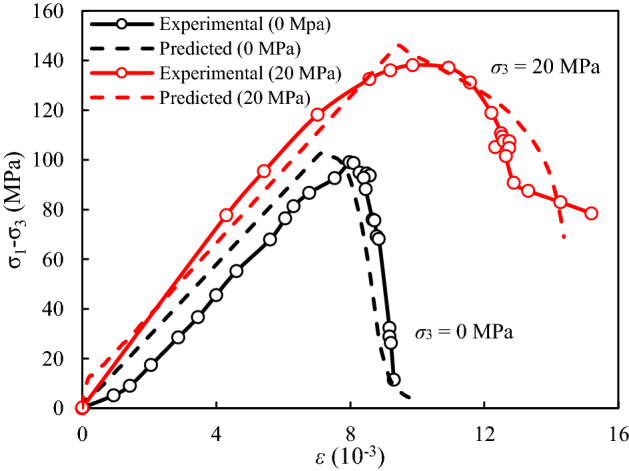
The complete stress–strain curves of sandstone samples (*β* = 0°) with different *σ*_3_ from experimental and predicted results.

Additionally, to predict the complete stress–strain curves, the piecewise-linear softening law was introduced to describe the post-peak strain-softening behavior of rock^35^. Take the triaxial compression test of sandstone specimens with β = 0° presented by Zhang et al.^32^ as an example. The complete stress–strain curves of sandstone samples (β = 0°) with different σ_3_ from experimental and predicted results are plotted in Fig. 9, from which the predicted stress–strain curves calculated by proposed model are basically consistent with the experimental results.

The modified Lade failure criterion describe the effect of intermediate principal stress on rock strength more correctly than M-C and Drucker-Prager criterion, and was governed by the most commonly used *c* and *φ* from the M-C failure criterion^24^, which enhances its attractiveness for applications in engineering field. However, the modified Lade failure criterion cannot accurately describe the non-linear mechanical behavior of rock under high temperature or high pressure due to its linear nature of the shear strength envelope. Also, the proposed criterion and model in this work have similar restriction.

## Conclusions

Due to the anisotropic characteristics appearing in the uniaxial or triaxial compressive test, the anisotropic modified Lade failure criterion is presented, which takes the effect of intermediate principal stress into account. The anisotropy of the rocks is considered by the variation of the parameters *c*_*β*_ and *φ*_*β*_ from the modified Lade criterion using an empirical equation. The correctness of the proposed criterion is verified by an extensive triaxial test database.

The incremental constitutive model of the anisotropic rocks is derived based on the modified Lade failure criterion. The DLL module is developed by using the VC++ program combined with the finite difference theory. The developed model is successfully validated based on different uniaxial and triaxial compressive experiments, which shows a good agreement between numerical and experimental results under various confining pressures.

## Supplementary Information

Supplementary Information.

## Data Availability

The datasets used and/or analysed during the current study available from the corresponding author on reasonable request.

## Abbreviations

DLL: Dynamic link library
M–C: Mohr–Coulomb
H–B: Hoek–Brown
UCS: Uniaxial compressive strength
PE: Percent error
COA: Coefficient of accordance
AAREP: Average absolute relative error percentage
