# Supplementary material for: Implementation of a constitutive model for anisotropic rocks based on modified Lade failure criterion
Source: Sci Rep. 2023 Feb 24;13:3210. doi: 10.1038/s41598-023-30257-z (PMC9958133; doi:10.1038/s41598-023-30257-z)
Supplement: Supplementary file 1 — Supplementary Information. [file 41598_2023_30257_MOESM1_ESM.docx]

**Appendix**

For the sake of completeness of presentation, the stress tensor invariants and the partial stress tensor invariants can be expressed as follows

|  | $\left\{\begin{matrix} I_{1}=\sigma^{\_} _{1}+\sigma^{\_} _{2}+\sigma^{\_} _{3} \\ I_{3}=\sigma^{\_} _{1}\sigma^{\_} _{2}\sigma^{\_} _{3} \\ J_{2}=\frac{1}{6}\left[(\sigma^{\_} _{1}-\sigma^{\_} _{2})^{2}+(\sigma^{\_} _{2}-\sigma^{\_} _{3})^{2}+(\sigma^{\_} _{3}-\sigma^{\_} _{1})^{2}\right] \\ J_{3}=\frac{1}{27}\left[(2\sigma^{\_} _{1}-\sigma^{\_} _{2}-\sigma^{\_} _{3})(2\sigma^{\_} _{2}-\sigma^{\_} _{1}-\sigma^{\_} _{3})(2\sigma^{\_} _{3}-\sigma^{\_} _{1}-\sigma^{\_} _{2})\right] \end{matrix}\right.$ | (42) |
| --- | --- | --- |

And the relationship between stress tensor and invariants can be given

|  | $\left\{\begin{matrix} I_{2}=\frac{1}{3}I_{1}^{2}-J_{2}, \\ I_{3}=J_{3}+\frac{1}{3}I_{1}I_{2}-\frac{2}{27}I_{1}^{3} \end{matrix}\right.$ | (43) |
| --- | --- | --- |

The corresponding stress Lode angle $\theta_{\overline{\sigma}}$ is

|  | $\theta_{\overline{\sigma}}=\frac{1}{3}\arcsin(-\frac{3\sqrt{3}J_{3}}{2J_{2}^{3}}), -\frac{\pi}{6}\leq\theta_{\overline{\sigma}}\leq\frac{\pi}{6}$ | (44) |
| --- | --- | --- |
